# Clinical and MRI remission in patients with nonradiographic axial spondyloarthritis who received long-term open-label adalimumab treatment: 3-year results of the ABILITY-1 trial

**DOI:** 10.1186/s13075-018-1556-5

**Published:** 2018-03-27

**Authors:** Désirée van der Heijde, Joachim Sieper, Walter P. Maksymowych, Robert G. Lambert, Su Chen, Maja Hojnik, Jaclyn K. Anderson, Aileen L. Pangan

**Affiliations:** 1 Leiden University Medical Center, PO Box 9600, Leiden, 2300 RC The Netherlands; 2 Charité Universitätsmedizin Berlin, Berlin, Germany; 3 University of Alberta, Edmonton, AB Canada; 4 AbbVie Inc., North Chicago, IL USA; 5 AbbVie Inc., Ljubljana, Slovenia

**Keywords:** Anti-TNF, Axial spondyloarthritis, Adalimumab

## Abstract

**Background:**

Adalimumab was effective in treating patients with nonradiographic axial spondyloarthritis (nr-axSpA) in the 12-week ABILITY-1 trial. We present long-term efficacy and safety results of adalimumab from the open-label ABILITY-1 extension, including the relationship between clinical and magnetic resonance imaging (MRI) remission and impact of sustained clinical remission on physical function.

**Methods:**

Patients received adalimumab 40 mg every other week or placebo for 12 weeks, then open-label adalimumab for up to 144 weeks. Clinical and safety data were collected through 3 years, and MRI data were collected until 2 years. Analyses were performed in the total population and subpopulation with positive MRI and/or elevated C-reactive protein (MRI/CRP-positive) at baseline. Clinical and MRI remission definitions included Ankylosing Spondylitis Disease Activity Score inactive disease (ASDAS ID; score < 1.3) and Spondyloarthritis Research Consortium of Canada (SPARCC) MRI score < 2 for sacroiliac joints (SIJs), spine, or both. Physical function was assessed using the Bath Ankylosing Spondylitis Functional Index.

**Results:**

Overall, 185 patients were included in the total population and 142 in the MRI/CRP-positive subpopulation; 65% and 68%, respectively, completed 3 years. Clinical, functional, and MRI improvements were similar and equally sustainable in both populations. At year 3, the percentages of patients in ASDAS ID in the MRI/CRP-positive subpopulation were 30%/33% (nonresponder imputation) and 46%/49% (observed) for those initially receiving adalimumab/placebo. At years 1 and 2, patients in ASDAS ID vs not had significantly greater improvements in SPARCC SIJ scores from baseline (*P* < 0.001). Among patients with baseline MRI scores ≥ 2 who achieved ASDAS ID at year 2, 44–68% also had MRI remission. Significantly more patients with sustained ASDAS ID through year 2 or 3 vs without achieved normal physical function (100% vs 48%; 100% vs 44%; both *P* < 0.001). No new safety concerns were observed.

**Conclusions:**

In the ABILITY-1 study of nr-axSpA, adalimumab therapy provided sustained clinical and functional improvements through 3 years, as well as suppression of MRI axial inflammation, which was greater in patients who achieved clinical remission. Sustained clinical remission was associated with increased attainment of normal physical function. The safety profile of adalimumab was consistent with prior studies.

**Trial registration:**

ClinicalTrials.gov, NCT00939003; registered on July 10, 2009.

**Electronic supplementary material:**

The online version of this article (10.1186/s13075-018-1556-5) contains supplementary material, which is available to authorized users.

## Background

Patients with axial spondyloarthritis (axSpA) include those with radiographic axSpA (also termed *ankylosing spondylitis* [AS]), which requires radiographic evidence of sacroiliitis according to the modified New York criteria [1], and those with nonradiographic axial spondyloarthritis (nr-axSpA) [2], who have similar signs and symptoms but without such radiographic changes. Magnetic resonance imaging (MRI) is increasingly used as an imaging tool in patients with axSpA [3, 4] for diagnosis, classification, and monitoring of disease activity [5–7]. Thus, there is a need to better understand the relationship between inflammation assessed by MRI and disease activity assessed clinically, as well as their impact on long-term physical function in patients with axSpA.

Therapeutic targets are emerging for axSpA. Clinical remission or inactive disease has been recommended as a major treatment target in the most recent updates of the Assessment of SpondyloArthritis international Society (ASAS)/European League Against Rheumatism management recommendations for axSpA and treat-to-target recommendations for spondyloarthritis [8, 9]. Once this goal is achieved, clinical remission should ideally be maintained over time. Ankylosing Spondylitis Disease Activity Score inactive disease (ASDAS ID) is the preferred definition of clinical remission [9]; however, more data on the long-term impact of remission status on physical function and quality of life are needed [8].

Treatment options for axSpA include nonsteroidal anti-inflammatory drugs (NSAIDs) and biologic disease-modifying antirheumatic drugs that target tumor necrosis factor (TNF)-α (i.e., anti-TNF agents) [10] or interleukin (IL)-17 (i.e., anti-IL-17) [11, 12]. Only anti-TNFs have proven efficacy in patients with nr-axSpA to date [8]. Adalimumab was effective for the treatment of patients with nr-axSpA in the ABILITY-1 trial [13]. Greater improvements in disease activity, physical function, and health-related quality of life, as well as reduction of sacroiliac joint (SIJ) and spine inflammation, based on MRI findings were demonstrated after 12 weeks of adalimumab therapy compared with placebo. Positive week 12 results were further supported by sustained clinical response and remission data through year 2 of the study [14].

This paper reports the final efficacy and safety data from the open-label extension (OLE) of the ABILITY-1 study in patients with nr-axSpA who received adalimumab for up to 3 years. Specifically, we evaluated the relationship between clinical and MRI remission, as well as the impact of sustained clinical remission on long-term physical function.

## Methods

### Patients

Patients were ≥ 18 years old with nr-axSpA and fulfilled the ASAS classification criteria for axSpA [2] but did not meet the modified New York criteria for AS [1]. Patients had active disease based on a Bath Ankylosing Spondylitis Disease Activity Index (BASDAI) score ≥ 4 (0–10 score) and total back pain score ≥ 4 on a 0- to 10-cm visual analogue scale (VAS; ≥ 40 on a 0- to 100-mm VAS). Patients must have also had an inadequate response or intolerance to at least one NSAID or a contraindication for NSAIDs. More detailed inclusion and exclusion criteria are published elsewhere [13].

### Study design

ABILITY-1 (ClinicalTrials.gov, NCT00939003) was a phase 3, multicenter, randomized, double-blind, placebo-controlled trial conducted at 37 sites in Australia, Europe, and North America. It was designed to evaluate the efficacy and safety of adalimumab in patients with nr-axSpA [13]. Data were collected from August 2009 through August 2013. Patients were randomized to subcutaneous adalimumab 40 mg every other week or placebo during a 12-week double-blind period, followed by a 144-week OLE in which patients received adalimumab 40 mg every other week for a total of 3 years of study participation. Study visits occurred at weeks 2 and 4, then every 4 weeks until week 28, every 8 weeks until week 68, every 12 weeks until week 140, and at week 156. The study was conducted with the approval of the appropriate institutional ethics review boards and with voluntary written informed patient consent, as well as in accordance with the International Conference on Harmonization good clinical practice guideline and the Declaration of Helsinki.

### Clinical and safety assessments

The efficacy and safety outcomes through week 12 of treatment were previously published [13]. Clinical disease activity was measured using the BASDAI and the Ankylosing Spondylitis Disease Activity Score (ASDAS) based on C-reactive protein (CRP). Criteria for clinical remission included ASDAS ID, defined as ASDAS < 1.3 [15], and ASAS partial remission (ASAS PR), defined as a score < 2 (0–10 scale) for each of the four components of the ASAS response criteria [16]. Sustained clinical remission was defined as meeting the remission criteria of interest (ASDAS ID) at all available study visits during the respective study period: year 2 (between weeks 52 and 104), year 3 (between weeks 104 and 156), and during both years 2 and 3 (between weeks 52 and 156). Clinical response is presented using ASAS20 and ASAS40, defined as ≥ 20% and ≥ 40% improvement, respectively, and an absolute improvement from baseline of ≥ 1 and ≥ 2 units, respectively, in at least three of the following four domains without worsening (defined as ≥ 20% worsening and net worsening from baseline ≥ 1 unit for ASAS20 and no worsening at all for ASAS40) in the remaining domain: patient global assessment of disease activity (0- to 10-cm VAS), total back pain (0- to 10-cm VAS), Bath Ankylosing Spondylitis Functional Index (BASFI; 0- to 10-cm VAS), and inflammation/morning stiffness (mean score of items 5 and 6 of the BASDAI [0- to 10-cm VAS]) [17]. Physical function was measured using BASFI [18]; normal/patient acceptable physical function was defined as BASFI ≤ 3 [19].

MRI of the SIJ and spine was performed at baseline and weeks 12, 52, and 104. Two central readers blinded to radiographs, treatment, and time point independently scored all MRIs of an individual patient, first scoring the SIJ using the Spondyloarthritis Research Consortium of Canada (SPARCC) method for SIJ (0–72) [20], followed by the SPARCC six-discovertebral unit method for spine (0–108) [21]. MRI remission was defined as a SPARCC score < 2 for the SIJ, spine, or both. Treatment-emergent adverse events (AEs) were defined as AEs that began or worsened after the first dose of study medication through 70 days after the last dose.

### Statistical methods

Clinical outcomes during the OLE were summarized in two ways: (1) by an observed case analysis of completers and (2) nonresponder imputation (NRI) for categorical variables or last observation carried forward (LOCF) analyses for continuous variables in the total efficacy population, which consisted of all randomized patients who received at least one dose of study drug (adalimumab or placebo). Subgroup analyses using the same methods were performed in patients with objective evidence of inflammation at baseline, defined as a SPARCC MRI score ≥ 2 for either the SIJ or spine or both, or elevated CRP (referred to as the *MRI/CRP-positive subpopulation*). In addition, key long-term clinical outcomes were also analyzed in the subgroup of patients without objective evidence of inflammation at baseline (referred to as the *MRI- and CRP-negative subpopulation*).

Mean changes from baseline in SPARCC MRI scores were calculated at weeks 52 and 104 for patients who achieved clinical remission at the same time points and compared with those who did not, using observed case analysis and analysis of variance. To assess the impact of sustained clinical remission on physical function, patients in the MRI/CRP-positive subpopulation with normal/abnormal physical function at baseline were grouped by achieving or not achieving sustained clinical remission during year 2, year 3, or throughout years 2 and 3 combined, and the proportions of patients with normal or abnormal physical function at years 2 and 3 (weeks 104 and 156) were compared by chi-square test (or Fisher’s exact test if ≥ 20% of the cells had expected counts ≤ 5).

The safety population consisted of all randomized patients who received at least one dose of adalimumab during the study. AEs were summarized as the number and percentage of patients experiencing AEs and number of events per 100 patient-years of adalimumab exposure using the Medical Dictionary for Regulatory Activities (MedDRA®) version 15.1 system organ classes and preferred terms.

## Results

### Patient disposition and characteristics

**Fig. 1:**
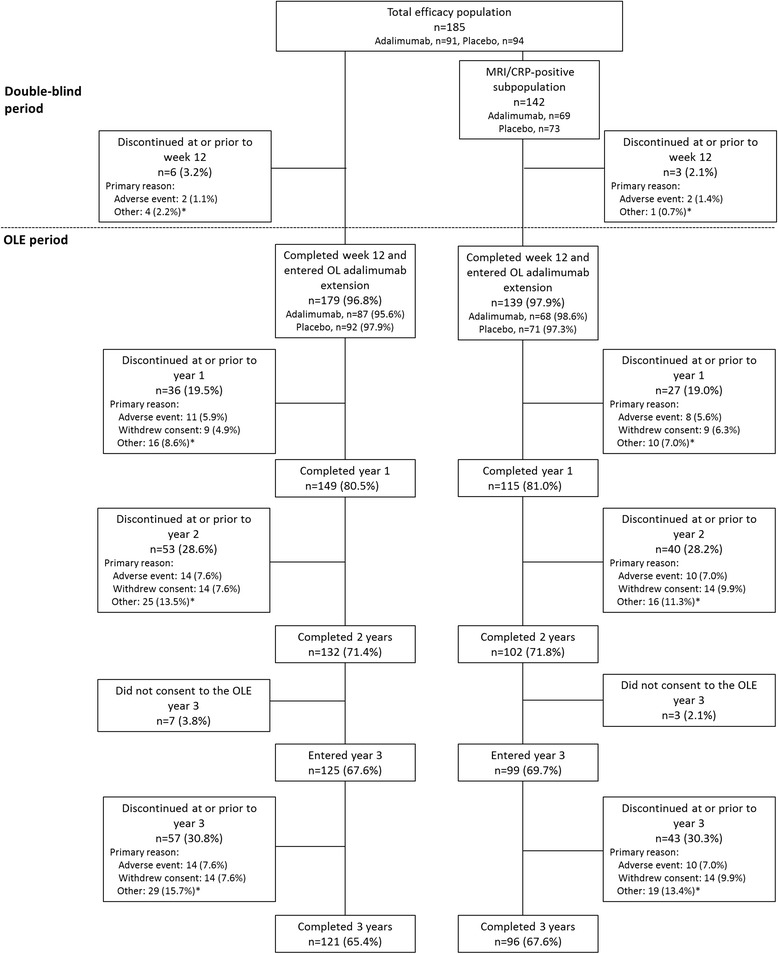
Patient disposition in the total efficacy and MRI/CRP-positive populations over 3 years. *Other reasons included lack of efficacy, pregnancy, investigator decision, patient moved, and protocol violation. OL Open label, OLE Open-label extension

Overall, 185 patients were included in the total efficacy population (adalimumab, *n* = 91; placebo, *n* = 94), and 142 patients were included in the MRI/CRP positive subpopulation (adalimumab, *n* = 69; placebo, *n* = 73) [13]; 42 patients had confirmed absence of MRI inflammation and normal CRP at baseline (MRI- and CRP-negative subpopulation); 1 patient who had normal CRP at baseline but missing baseline MRI assessment was excluded from the MRI- and CRP-negative subpopulation analyses but was included in the baseline data (Table 1). A total of 179 patients (adalimumab, *n* = 87 [96%]; placebo, *n* = 92 [98%]) from the total efficacy population and 139 patients (adalimumab, *n* = 68 [99%]; placebo, *n* = 71 [97%]) from the MRI/CRP-positive subpopulation completed the double-blind period and entered the OLE (Fig. 1). Similar proportions of patients in the total efficacy population (81%, 71% and 65%) and MRI/CRP-positive subpopulation (81%, 72%, and 68%) completed years 1, 2, and 3 of the OLE, respectively (Fig. 1). In the MRI- and CRP-negative subgroup, 57% of patients completed year 3.

**Table 1:** Baseline demographics and disease characteristics

Characteristic, mean (SD)	Total efficacy population *(N* = 185)	MRI/CRP-positive subpopulation *(n* = 142)	MRI- and CRP-negative subpopulation *(n* = 43)^a^
Female, n (%)	101 (54.6)	77 (54.2)	24 (55.8)
Age, years	38.0 (10.8)	38.3 (11.1)	37.2 (10.1)
Symptom duration, years	10.0 (8.9)^b^	10.5 (9.4)^c^	8.3 (6.6)^d^
HLA-B27 positive, n (%)	145 (78.4)	114 (80.3)	31 (72.1)
BASDAI (0–10)	6.5 (1.5)	6.4 (1.5)	6.7 (1.5)
ASDAS (0–10)	3.3 (0.8)^e^	3.4 (0.8)^f^	2.9 (0.6)^d^
Patient Global Assessment of Disease Activity (0–10 cm VAS)	6.8 (1.8)	6.8 (1.9)	6.8 (1.8)
Physician Global Assessment of Disease Activity (0–10 cm VAS)	5.7 (1.6)^g^	5.6 (1.6)	6.1 (1.7)^h^
Total back pain (0–10 cm VAS)	6.9 (1.8)	6.8 (1.8)	7.3 (1.5)
BASFI (0–10)	4.7 (2.1)^g^	4.7 (2.2)^i^	4.8 (2.0)
Inflammation/morning stiffness^j^ (0–10 cm VAS)	6.6 (2.0)	6.6 (2.1)	6.7 (1.7)
Elevated baseline CRP,^k^ n (%)	48 (33.6)^l^	48 (46.2)^m^	0
CRP, ^k^ mg/L	7.2 (11.0)	9.0 (12.0)	1.4 (1.1)
SPARCC MRI score, SIJ	4.9 (9.7)^n^	6.3 (10.7)^c^	0.4 (0.5)^h^
SPARCC MRI score, spine	4.3 (5.8)^o^	5.5 (6.1)^p^	0.5 (0.6)^h^
HAQ-S (0–3)	1.0 (0.6)	1.0 (0.5)	1.1 (0.6)
BASMI linear (0–10)	2.7 (1.2)^g^	2.7 (1.3)^i^	2.7 (1.1)

*Abbreviations: ASDAS* Ankylosing Spondylitis Disease Activity Score, *BASDAI* Bath Ankylosing Spondylitis Disease Activity Index, *BASFI* Bath Ankylosing Spondylitis Functional Index, *BASMI* Bath Ankylosing Spondylitis Metrology Index, *CRP* C-reactive protein, *HAQ-S* Health Assessment Questionnaire for the Spondyloarthropathies, *HLA-B27* human leukocyte antigen B27, *MRI* Magnetic resonance imaging, *SPARCC* Spondyloarthritis Research Consortium of Canada, *VAS* Visual analogue scale

^a^The baseline data for the MRI- and CRP-negative subpopulation includes 42 patients with confirmed absence of MRI inflammation and normal CRP at baseline and 1 patient with normal CRP but missing baseline MRI,^b^*n* = 180,^c^*n* = 140,^d^*n* = 40,^e^*n* = 178,^f^*n* = 138,^g^*n* = 184,^h^*n* = 42,^i^*n* = 141,^j^Mean of BASDAI questions 5 and 6,^k^Based on either standard or high-sensitivity CRP values,^*l*^*n* = 143,^m^*n* = 104,^n^*n* = 182,^o^*n* = 181,^p^*n* = 139

The demographic and clinical characteristics were similar between the total efficacy population and the MRI/CRP-positive subpopulation (Table 1). Slightly more than half were female (55% and 54%, respectively), mean age was 38 years, and mean symptom duration was approximately 10 years. Patients had similar disease activity and impairment in physical function (Table 1). Among the 43 patients in the MRI- and CRP-negative population, including the 1 patient with normal CRP but missing MRI at baseline, mean ASDAS was slightly lower (most likely due to lower CRP), symptom duration was shorter, and a smaller proportion of patients were human leukocyte antigen (HLA)-B27-positive at baseline compared with the MRI/CRP-positive subpopulation (Table 1).

### Maintenance of clinical and functional improvements through 3 years

**Fig. 2:**
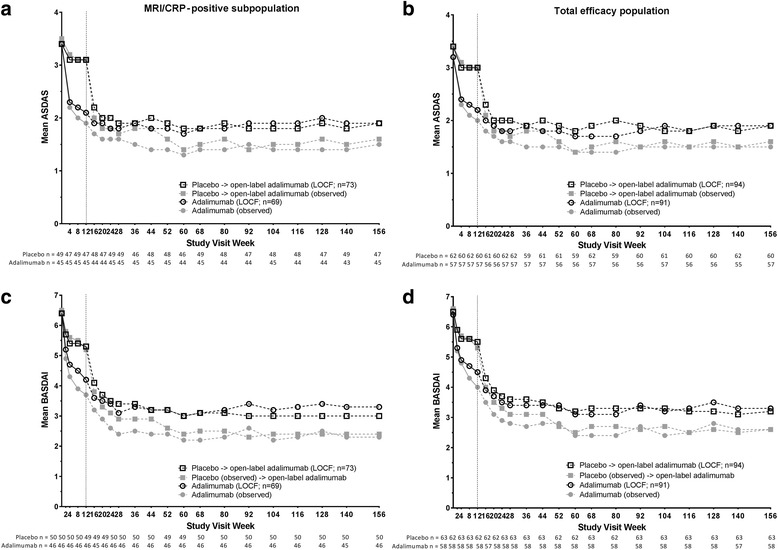
Mean ASDAS and BASDAI scores over 3 years. Mean ASDAS in the (**a**) MRI/CRP-positive subpopulation and (**b**) total efficacy population and mean BASDAI in the (**c**) MRI/CRP-positive subpopulation and (**d**) total efficacy population. Observed data (closed gray symbols) and LOCF data (open black symbols) through 3 years of OLE. Dashed line represents the start of the open-label adalimumab period; n values below the x-axis are for observed data at each visit, and n values in the figure legend are for the LOCF data. ASDAS Ankylosing Spondylitis Disease Activity Score, BASDAI Bath Ankylosing Spondylitis Disease Activity Index, CRP C-reactive protein, LOCF last observation carried forward, MRI magnetic resonance imaging

The improvements in ASDAS and BASDAI with adalimumab treatment were similar and sustained in the MRI/CRP-positive subpopulation and the total efficacy population (Fig. 2). At week 156, mean (SD) ASDAS was 1.9 (1.2) and BASDAI was 3.3 (2.7) in the initial adalimumab group of the MRI/CRP-positive subpopulation (LOCF) (Figs. 2a and c); the mean (SD) changes from baseline to week 156 were − 1.4 [1.3] for ASDAS and − 3.1 [2.6] for BASDAI. Similar improvements in ASDAS and BASDAI were noted for the MRI- and CRP-negative subpopulation; at week 156, the mean (SD) ASDAS was 1.8 (1.0) and BASDAI was 3.7 (2.6), and the mean (SD) changes from baseline to week 156 were − 1.1 (1.0) for ASDAS and − 2.9 (2.7) for BASDAI (LOCF).

In the MRI/CRP-positive subpopulation, ASAS40 response was achieved by approximately half (NRI) or two-thirds (observed) of the patients through years 1, 2, and 3 (Additional file 1: Figure S1). Similar ASAS40 responses were observed in the total efficacy population, whereas ASAS40 response was lower in the MRI- and CRP-negative subpopulation (Additional file 1: Figure S1).

**Fig. 3:**
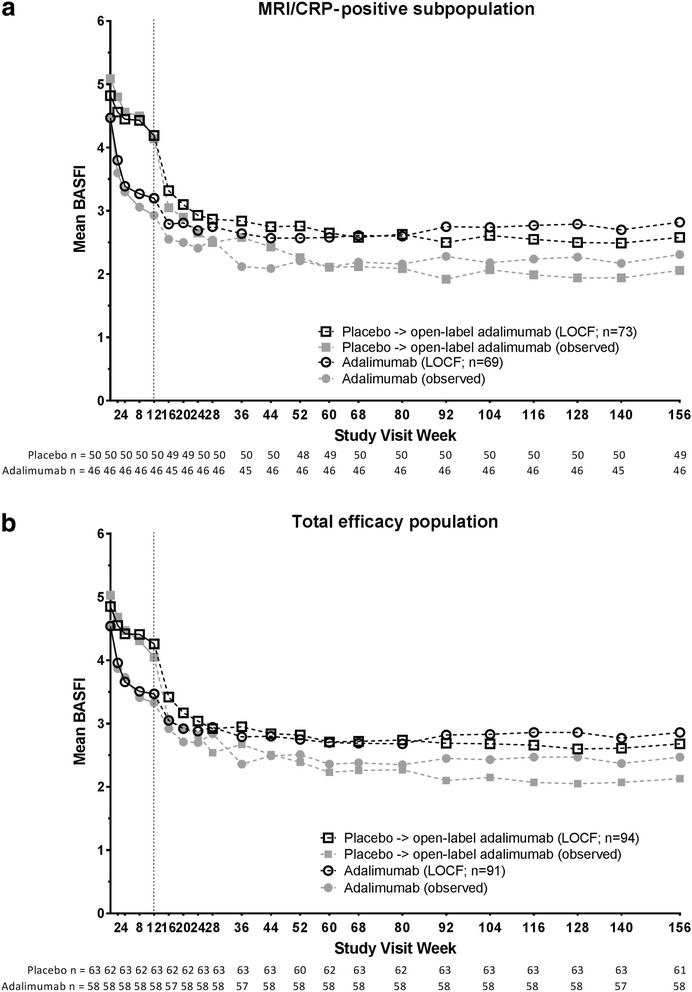
Mean BASFI scores over 3 years. Mean BASFI in the (**a**) MRI/CRP-positive subpopulation and (**b**) total efficacy population. Observed data (closed gray symbols) and LOCF data (open black symbols) through 3 years of OLE. Dashed lines represent the start of the open-label adalimumab period; n values below the x-axis are for observed data at each visit, and n values in the figure legend are for the LOCF data. BASFI Bath Ankylosing Spondylitis Functional Index, CRP C-reactive protein, LOCF last observation carried forward, MRI magnetic resonance imaging

The improvements in physical function were also sustained with adalimumab treatment through 3 years. In the MRI/CRP-positive subpopulation, the mean (SD) BASFI score at week 156 was 2.8 (2.6) and mean (SD) change from baseline to week 156 was − 1.7 (2.2) in the initial adalimumab group (LOCF) (Fig. 3a). Improvements in physical function were comparable in the total efficacy population (Fig. 3b) and in the MRI- and CRP-negative subpopulation (mean [SD] BASFI at week 156, 3.0 [2.5]; mean [SD] change from baseline to week 156, − 1.9 [2.5]; LOCF).

### Maintenance of MRI improvements through 2 years

There was a significant reduction in both SPARCC MRI SIJ and spinal inflammation scores from baseline to 1 and 2 years in the MRI/CRP-positive subpopulation and total efficacy population (all *P* < 0.001). Among patients in both populations who had both year 1 and year 2 MRI scans available, sustained improvements in MRI inflammation were evident through year 2 (Table 2).

**Table 2:** Mean baseline and changes from baseline in SPARCC MRI SIJ and spine scores

Mean (95% CI)	MRI/CRP-positive subpopulation		Total efficacy population	
	SPARCC MRI SIJ (*n* = 102)	SPARCC MRI spine (*n* = 101)	SPARCC MRI SIJ (*n* = 131)	SPARCC MRI spine (*n* = 130)
Baseline	6.2 (4.1–8.2)	4.3 (3.3–5.4)	5.0 (3.3–6.6)	3.5 (2.6–4.3)
Change from baseline to:				
Year 1	− 5.0 (− 6.9 to − 3.1)	− 1.9 (− 2.8 to − 1.0)	− 3.9 (− 5.5 to − 2.4)	− 1.3 (− 2.1 to − 0.5)
Year 2	− 4.8 (− 6.9 to – 2.8)	− 2.0 (− 2.9 to − 1.1)	− 3.8 (− 5.4 to − 2.2)	− 1.4 (− 2.2 to − 0.7)

*Abbreviations: CRP* C-reactive protein, *MRI* Magnetic resonance imaging, *SIJ* Sacroiliac joint, *SPARCC* Spondyloarthritis Research Consortium of Canada

Data for patients in the MRI/CRP-positive subpopulation and total efficacy population with MRI scans available at all time points

### Clinical and MRI remission achievement and agreement

**Fig. 4:**
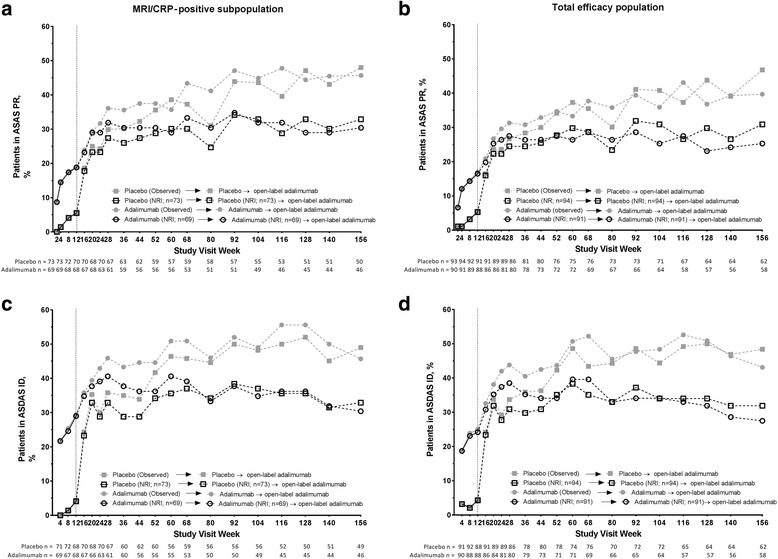
Proportion of patients achieving clinical remission over 3 years. ASAS partial remission in the (**a**) MRI/CRP-positive subpopulation and (**b**) total efficacy population and ASDAS ID (ASDAS < 1.3) in the (**c**) MRI/CRP-positive subpopulation and (**d**) total efficacy population. Observed data are shown with closed gray symbols and NRI data with open black symbols; the dashed line represents the start of the open-label adalimumab period. n Values below the x-axis are for observed data at each visit. n Values in the figure legend are for NRI data; patients who permanently discontinued at or prior to week 12 (double-blind phase) were excluded from open-label phase analyses (MRI/CRP-positive subpopulation, *n* = 3; total efficacy population, *n* = 6). ASAS Assessment of SpondyloArthritis international Society, ASDAS Ankylosing Spondylitis Disease Activity Score, CRP C-reactive protein, ID Inactive disease,  MRI Magnetic resonance imaging, NRI Nonresponder imputation

Clinical remission rates were maintained through 3 years in both the MRI/CRP-positive subpopulation and the total efficacy population (Fig. 4). In the MRI/CRP-positive subpopulation among patients receiving adalimumab throughout 3 years, 32% and 30% of patients achieved ASAS PR and 35% and 30% achieved ASDAS ID at years 2 and 3, respectively, using NRI analysis. The rates were 45% and 46% for ASAS PR and 49% and 46% for ASDAS ID at years 2 and 3, respectively, by observed data (Fig. 4a and c). In the MRI- and CRP-negative subpopulation, 14% and 17% of patients achieved ASAS PR (NRI) at years 2 and 3, respectively, and 29% and 24%, respectively, achieved ASDAS ID (NRI).

**Fig. 5:**
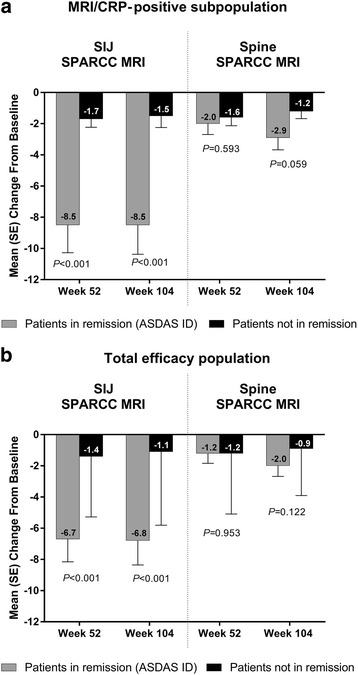
Change from baseline in SPARCC MRI scores among patients who achieved clinical remission. Mean (SE) change from baseline in the SIJ and spine SPARCC MRI scores in patients who achieved ASDAS ID vs those who did not in the (**a**) MRI/CRP-positive subpopulation and (**b**) total efficacy population at years 1 and 2. Observed data. *P* values for comparison of patients in remission vs patients not in remission from analysis of variance. ASDAS ID Ankylosing Spondylitis Disease Activity Score inactive disease, CRP C-reactive protein, MRI Magnetic resonance imaging, SIJ Sacroiliac joint, SPARCC Spondyloarthritis Research Consortium of Canada

The mean SIJ SPARCC MRI scores decreased significantly more (*P* < 0.001) from baseline to years 1 and 2 in patients in clinical remission (ASDAS ID) than in those not in ASDAS ID at years 1 and 2 (Fig. 5a and b). The reductions from baseline in the spine SPARCC MRI scores were not significantly different between patients who achieved clinical remission and those who did not.

Among patients in the MRI/CRP-positive subpopulation who had positive baseline MRI for the SIJ, spine, or both, MRI remission in the respective location was achieved by 65%, 48%, and 41% of patients at year 1, and by 71%, 52%, and 50% at year 2 (observed). Among patients in clinical remission (ASDAS ID) who had positive baseline MRI for the SIJ, spine, or both, MRI remission was achieved by 73%, 53%, and 40% of patients, respectively, at year 1, and by 68%, 68%, and 44% at year 2 (Additional file 1: Figure S2a). Similarly, among patients in MRI remission who had positive baseline MRI for the SIJ, spine, or both, clinical remission (ASDAS ID) was achieved by 67%, 50%, and 67%, respectively, at year 1, and by 58%, 63%, and 62% at year 2 (Additional file 1: Figure S2b). Of the patients who had positive baseline MRI for the SIJ, spine, or both, and who did not achieve clinical remission, 56%, 43%, and 44% had MRI remission of the SIJ, spine, or both, respectively, at year 1, as did 74%, 35%, and 56% at year 2.

### Sustained clinical remission and its impact on long-term physical function

**Fig. 6:**
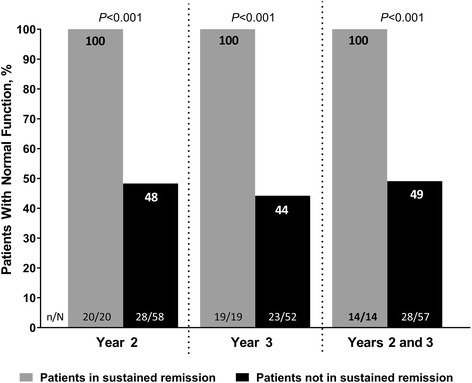
Patients in sustained remission achieving normal physical function at years 2 and 3. Analysis of patients in the MRI/CRP-positive subpopulation with abnormal physical function at baseline (normal function defined as BASFI ≤ 3). Sustained clinical remission was defined as achieving ASDAS < 1.3 at all available visits during year 2 (from week 52 to week 104), during year 3 (from week 104 to week 156), and during both years 2 and 3 (from week 52 to week 156). ASDAS Ankylosing Spondylitis Disease Activity Score, BASFI Bath Ankylosing Spondylitis Functional Index, CRP C-reactive protein, MRI Magnetic resonance imaging

Sustained clinical remission (defined as ASDAS ID at all time points) was achieved by 34 of 142 (24%) patients in year 2 (between weeks 52 and 104), 32 of 142 (23%) patients in year 3 (between weeks 104 and 152), and 25 of 142 (18%) patients through both years 2 and 3 (between weeks 52 and 156) in the MRI/CRP-positive subpopulation (NRI analysis). At baseline, 102 patients in the MRI/CRP-positive subpopulation had abnormal physical function (BASFI score > 3); among these patients, normal function was achieved by 48 of 78 (62%) patients at year 2 and 42 of 71 (59%) patients at year 3. Among patients with abnormal physical function at baseline, all patients in sustained clinical remission during year 2, year 3, or through both years 2 and 3 achieved normal function (20 of 20, 19 of 19, and 14 of 14, respectively) compared with less than half of the patients not in sustained clinical remission (28 of 58 [48%], 23 of 52 [44%], and 28 of 57 [49%], respectively; all *P* < 0.001) (Fig. 6). All patients with normal physical function at baseline (*n* = 39) maintained normal function at years 2 and 3, regardless of being in remission or not.

### Safety

Over 400 patient-years of adalimumab exposure were included in the long-term safety evaluation (Table 3). The types and rates of AEs in the MRI/CRP-positive subpopulation were similar to those in the overall study population. No incidents of malignancy, vasculitis, demyelinating disease, or reactivation of hepatitis B were reported.

**Table 3:** Rates of adverse events of interest through 3 years of treatment

AE	Overall safety population (*n* = 190; 412.2 patient-years)		MRI/CRP-positive safety subpopulation (*n* = 145; 320.9 patient-years)	
	No. (%)	Events/100 patient-years	No. (%)	Events/100 patient-years
Serious AEs	33 (17.4)	10.9	25 (17.2)	9.7
Serious infection	8 (4.2)	2.4	6 (4.1)	2.2
Tuberculosis	1 (0.5)	0.2	1 (0.7)	0.3
Lupus-like reaction and systemic lupus erythematosus	1 (0.5)	0.2	1 (0.7)	0.3
Pulmonary embolism	1 (0.5)	0.2	1 (0.7)	0.3
New-onset psoriasis	2 (1.1)	0.5	1 (0.7)	0.3
Hepatic-related AEs	2 (1.1)	0.5	1 (0.7)	0.3
Diverticulitis	1 (0.5)	0.2	1 (0.7)	0.3
Death	2 (1.1)	0.5	2 (1.4)	0.6

*Abbreviations: AE* Adverse event, *CRP* C-reactive protein, *MRI* Magnetic resonance imaging

Includes all patients who received at least one dose of adalimumab, including patients who were excluded from the efficacy analyses because of noncompliance at the investigative site

One patient experienced a lupus-like reaction during the OLE, presenting with facial flushing and joint pain associated with a positive antinuclear antibody and anti-double-stranded DNA. Adalimumab was discontinued because of the event, and symptoms improved. One patient with a family history of deep vein thrombosis (DVT), who was receiving hormonal birth control and had a prior event of DVT during the study, developed a pulmonary embolism. The patient discontinued the hormonal contraceptive device and was able to restart open-label adalimumab treatment after a temporary interruption.

There were two deaths. One was a suicide of a man with a history of heavy alcohol use and anxiety; his death occurred 40 days after discontinuation of open-label adalimumab. Another patient died of cardiorespiratory failure related to recreational opiate toxicity during open-label adalimumab treatment.

## Discussion

The results of the long-term extension of the ABILITY-1 study demonstrated sustained clinical and functional improvements through 3 years of adalimumab therapy in patients with nr-axSpA. Furthermore, there was a sustained reduction in MRI axial inflammation, which was significantly greater in the SIJ of patients who achieved clinical remission than in those not in remission. Sustained clinical remission was associated with attainment of normal physical function.

In contrast to AS, there is still limited understanding of the disease course and appropriate long-term management of patients with nr-axSpA. The present work adds to the body of evidence that clinical and MRI improvements can be maintained with long-term anti-TNF therapy in patients with nr-axSpA classified by ASAS criteria [22, 23]. Further, it provides important insights into the relationship between clinical and MRI remission in nr-axSpA, as well as the significance of sustained clinical remission for achieving normal physical function.

We evaluated the efficacy outcomes separately in the total efficacy population (*n* = 185) and subpopulation of patients with objective signs of inflammation at baseline (MRI/CRP-positive subpopulation; *n* = 142) for whom anti-TNF therapy, including adalimumab, is currently approved. No major differences were observed in the results, with the MRI/CRP-positive subpopulation representing 77% of the total efficacy population. For completeness, we also provided information for the MRI- and CRP-negative subpopulation.

Throughout the OLE, approximately two-thirds (as observed) or nearly half (NRI) of the patients in the MRI/CRP-positive subpopulation achieved ASAS40 response. Almost half (observed) or around one-third (NRI) of patients were in clinical remission measured as either ASAS PR or ASDAS ID at the end of the study. Furthermore, sustained clinical remission, defined as achieving ASDAS ID at all available visits, was seen in nearly one-fourth of patients through each of year 2 and year 3, with 18% of patients in sustained ASDAS ID throughout both years 2 and 3.

Although the clinical improvements observed in the small MRI- and CRP-negative subpopulation (*n* = 42) were generally smaller than in the MRI/CRP-positive subpopulation, it appears that some patients without objective evidence of inflammation at baseline responded to adalimumab treatment and maintained clinical improvements through 3 years. However, interpretation should be done with caution because of the small number of patients in this group.

MRI scans taken at 1 and 2 years showed a significant and sustained suppression of inflammation in both the SIJ and spine. An interesting observation was an apparent better responsiveness of the MRI inflammation in the SIJ than in the spine to adalimumab treatment. The reductions in the mean SPARCC MRI scores were relatively larger, and a higher proportion of patients achieved MRI remission (SPARCC MRI score < 2) in the SIJ than in the spine among patients with positive MRI at baseline; SIJ vs spine MRI remission rates were 65% vs 48% and 71% vs 52% at years 1 and 2, respectively. There also seemed to be a better numerical agreement between clinical and MRI remission in the SIJ than in the spine. Among patients with positive baseline MRI, a larger proportion of patients in clinical remission (ASDAS ID) reached MRI remission in the SIJ than in the spine at year 1 (73% vs 53%). Furthermore, significantly greater mean improvements from baseline were observed at years 1 and 2 for the SIJ SPARCC MRI scores in patients achieving ASDAS ID than in those not in ASDAS ID. The differences between patients in ASDAS ID vs those not in ASDAS ID were not significant for the spine SPARCC MRI scores.

Of note, the baseline MRI scores were numerically greater in the SIJ than in the spine, especially in the MRI/CRP-positive subpopulation, in spite of the mean symptom duration of around 10 years in this nr-axSpA population. It is generally believed that axial inflammation starts in the SIJ and then involves the spine in axSpA [24], but there was still considerable inflammation in the SIJ in this nr-axSpA study population. Another possible explanation for the somewhat lower effect on the spine MRI scores is that not all the lesions detected in the spine were specific for axSpA [25] or were responsive to anti-TNF treatment. It is also interesting that in the EMBARK study with etanercept in nr-axSpA, more patients achieved MRI remission in the spine than in SIJ after 2 years in a cohort with much shorter symptom duration (mean 2.4 years) [23]. However, the differences between the proportions of patients achieving either remission in the SIJ or spine were not significant between those with or without sustained ASDAS ID or low disease activity in that study. The definitions of MRI remission were less stringent, especially for the spine, in the EMBARK study than in the ABILITY-1 study [23].

A discordance between clinical and MRI disease activity in axSpA has been noted before [26–28]. In our study, only 40% and 44% of patients with positive baseline MRI who achieved ASDAS ID also attained overall MRI remission (both in SIJ and spine) at years 1 and 2, respectively. Numerically more patients achieving overall MRI remission among those with positive baseline MRI also met ASDAS ID criteria at years 1 and 2 (67% and 62%, respectively). Currently, clinical and not MRI remission is suggested as a treatment target [8, 9] because the impact of persistent MRI inflammation in the SIJ or spine on long-term physical function or structural progression in patients with axSpA who are in clinical remission is unknown. Further studies are needed in this area.

Evidence that has accumulated over the past few years in AS suggests a direct relationship of clinical disease activity with physical function and syndesmophyte formation [29–31]. The long-term impact of achieving and maintaining clinical remission in nr-axSpA has not previously been evaluated. Our results demonstrate the importance of sustained clinical remission (defined as ASDAS ID) for achieving normal physical function over time. All patients in sustained clinical remission achieved normal physical function at years 2 and 3, as compared with only 44–49% of patients not in sustained clinical remission. All patients with normal physical function at baseline maintained it until the end of the OLE with adalimumab, regardless of achieving sustained clinical remission or not. The total number of patients with normal physical function at baseline was small, however, and no definite conclusions can be drawn on the importance of sustained clinical remission for the maintenance of normal physical function. It also needs to be noted that our definition of sustained clinical remission was very strict, requiring ASDAS ID at all study visits. Our findings lend further support to ASDAS ID as a clinically meaningful definition of clinical remission [32]. They also substantiate the treat-to-target recommendation that once the target of clinical remission is achieved, remission should ideally be maintained throughout the patient’s disease course [9].

It is known that the extent of structural damage along with the degree of inflammation measured by disease activity determine physical function in AS [28, 29]. Limitations of our analyses include the lack of assessment of structural damage, which made it impossible to correlate sustained clinical remission or MRI remission with structural progression. MRI was also not performed at year 3, limiting the analyses of MRI findings to the years 1 and 2 time points. Safety data collected over 3 years did not reveal new safety concerns in nr-axSpA compared with the known safety profile of adalimumab in other immune-mediated inflammatory diseases, particularly in AS [33].

## Conclusions

Treatment of patients with nr-axSpA with adalimumab over 3 years demonstrated sustained clinical remission in a substantial proportion of patients, which was associated with the achievement or maintenance of normal physical function. Clinical remission and remission based on MRI were only partly concordant. Together with the safety data from the ABILITY-1 study, our findings support the favorable benefit-risk profile of long-term adalimumab therapy in patients with nr-axSpA.

## Additional file

Additional file 1: **Figures S1 and Figure S2.** Additional data. (DOCX 1105 kb)

## Abbreviations

AE: Adverse event
AS: Ankylosing spondylitis
ASAS: Assessment of SpondyloArthritis international Society
ASDAS: Ankylosing Spondylitis Disease Activity Score
axSpA: Axial spondyloarthritis
BASDAI: Bath Ankylosing Spondylitis Disease Activity Index
BASFI: Bath Ankylosing Spondylitis Functional Index
BASMI: Bath Ankylosing Spondylitis Metrology Index
CRP: C-reactive protein
DVT: Deep vein thrombosis
HAQ-S: Health Assessment Questionnaire for the Spondyloarthropathies
HLA-B27: Human leukocyte antigen B27
ID: Inactive disease
IL: Interleukin
LOCF: Last observation carried forward
MedDRA: Medical Dictionary for Regulatory Activities
MRI: Magnetic resonance imaging
nr-axSpA: Nonradiographic axial spondyloarthritis
NRI: Nonresponder imputation
NSAID: Nonsteroidal anti-inflammatory drug
OLE: Open-label extension
PR: Partial remission
SIJ: Sacroiliac joint
SPARCC: Spondyloarthritis Research Consortium of Canada
TNF: Tumor necrosis factor
VAS: Visual analogue scale
