# Supplementary material for: Clinical and MRI remission in patients with nonradiographic axial spondyloarthritis who received long-term open-label adalimumab treatment: 3-year results of the ABILITY-1 trial
Source: Arthritis Res Ther. 2018 Mar 27;20:61. doi: 10.1186/s13075-018-1556-5 (PMC5870399; doi:10.1186/s13075-018-1556-5)
Supplement: Supplementary file 1 — Figures S1 and Figure S2. Additional data. (DOCX 1105 kb) [file 13075_2018_1556_MOESM1_ESM.docx]

**Figure S1.** Percentage of patients in the total efficacy, MRI/CRP positive and MRI&CRP negative populations achieving ASAS20 and ASAS40 responses at years 1, 2, and 3 of the study. Solid bars, NRI (n=185, n=142, and n=42); hatched bars, observed case analysis (n=150, 138, and 122 at years 1, 2, and 3, respectively, for the total efficacy population; n=116, 107, and 97 for the MRI/CRP positive subpopulation; n=33, 30, and 24 for the MRI&CRP negative subpopulation). ASAS, assessment of spondyloarthritis international Society; CRP, C-reactive protein; MRI, magnetic resonance imaging; NRI, nonresponder imputation.

**Figure S2.** (A) Percentage of patients with MRI remission in the SIJ, spine, or both (SPARCC MRI scores <2) among patients who had baseline SPARCC MRI scores ≥2 for the specified location and who were in clinical remission (ASDAS inactive disease; ASDAS <1.3) at year 1 or 2. (B) Percentage of patients in clinical remission (ASDAS inactive disease) among patients in mri remission (SPARCC MRI scores <2) at year 1 or 2 who had baseline sacroiliac joint and/or spine SPARCC MRI scores ≥2. ASDAS, Ankylosing Spondylitis Disease Activity Score; MRI, magnetic resonance imaging; SIJ, sacroiliac joint; SPARCC, Spondyloarthritis Research Consortium of Canada.
